# Optimization Design Method of a New Stabilized Platform Based on Missile-borne Semi-Strap-down Inertial Navigation System

**DOI:** 10.3390/s18124412

**Published:** 2018-12-13

**Authors:** Jie Li, Zhengyao Jing, Xi Zhang, Jiayu Zhang, Jinqiang Li, Shiyao Gao, Tao Zheng

**Affiliations:** 1 Key Laboratory of Instrumentation Science & Dynamic Measurement, Ministry of Education, North University of China, Taiyuan 030051, China; s1706154@163.com (Z.J.); Zhangxi@nuc.edu.cn (X.Z.); 18734196406@163.com (J.Z.); S1706147@st.nuc.edu.cn (J.L.); S1706072@st.nuc.edu.cn (S.G.); 2 National Key Laboratory for Electronic Measurement Technology, North University of China, Taiyuan 030051, China; 3 Key Laboratory of Biomimetic Robots and Systems, Beijing Institute of Technology, Ministry of Education, Beijing 100081, China; 18634409496@163.com

**Keywords:** stabilized platform, minimum roll angular rate, lift force, navigation accuracy

## Abstract

At present, existing wide range Micro-Electro-Mechanical-Systems (MEMS) inertial sensors have relatively lower precision and direct measurement of the missile’s high-rotation motion inevitably uses a large-range sensor. To achieve high-precision navigation, this paper proposes a novel Semi-strap-down Stabilized Platform (SSP) based on the Missile-borne Semi-Strap-down Inertial Navigation System, which is used to mount sensors and lowers sensor range requirements through isolating the high-rotational motion of missile. First, the author innovatively puts forward a dynamic model under missile-borne environment, then analyses the influence of SSP quality on the range of gyro according to the dynamic model of the SSP. Finally, when the angle of attack of the missile is 2°, the best quality of the SSP with minimum roll angular rate amplitude was calculated through the Runge-Kutta method and the mass gradient control method. Experiments have been carried out by using a high-precision, tri-axial flight simulation turntable to validate the viability of the method. Experiments show that under the same conditions, the angular velocity of the new optimized SSP with the best quality design is reduced to 1/3 of the unoptimized SSP, and the measured roll angle error is reduced to 60% of the unoptimized measurement. The results indicate that the novel SSP has better performance segregating the high-speed rotational motion, and provides theoretical guidance for the high-precision small-range sensor instead of the low-precision wide-range sensor. In addition, the first proposed SSP quality selection method creates a new idea for the improvement of the positioning accuracy in the missile-borne environment.

## 1. Introduction

The high-speed-rotation and high-overload missile, as a kind of common missile, plays an essential role in modern military warfare [1,2]. Furthermore, the high-precision measurement of high-speed rotation missile attitude information is the key technology of guidance and precision strike, which is the main development trend of conventional high-speed rotation missile guidance [3,4]. Therefore, the implementation of accurate navigation and positioning for high-rotation and high-overload missiles is a key technology that needs an urgent breakthrough [5,6]. The data shows that the initial velocity of the missile launch is about 550 m/s, and its overload is up to 10,000 g, which makes most types of sensors not work properly. As a common device in inertial navigation, Micro-Electro-Mechanical-System (MEMS) sensors are widely used in navigation guidance because of their small size, low cost, and strong anti-overload capability [7]. Therefore, we choose Miniature Inertial Measurement Unit (MIMU) sensors to measure velocity, position, and attitude information. However, the common Strap-down Inertial Navigation System (SINS) is not suitable for high-dynamic, high-spinning, and high-overload missiles, due to the fact that the accelerometer and the gyroscope have poor anti-overload capability without protection, and the range of sensors used in the SINS is too large, resulting in a decrease in accuracy [8]. In this case, different from traditional measurement methods, the concept of Semi-Strapdown Inertial Navigation System (SSINS) is proposed by Key Laboratory of Instrumentation Science & Dynamic Measurement [9,10]. A mechanical structure, the Semi-strap-down Stabilized Platform (SSP) is a key part of SSINS, its main function is to reduce the range of sensors required and resist overload [11]. Therefore, we should further analyze its principle and design its structure, in order to achieve the optimal choice of sensor range, and improve the accuracy of navigation solution. 

In recent years, the measurement of missile attitude has attracted amount of attention. Raúl de Celis et al. proposed an approach for guidance of high rate spinning ballistic rockets, which is based on an innovative hybridization between GNSS/accelerometer and semi-active laser quadrant photo-detector [12]. In [12], the fusion scheme is studied, and it can precisely measure the attitude of the high-rotation missile, however GNSS cannot be completely independent and is not suitable for high overload environments. In Li’s paper, a method based on photoelectric theodolite to measure the roll angle of ammunition is proposed [13]. This method can precisely measure the roll angle of ammunition, applicable to measure the roll angle of low-dynamic missile. In Beijing Institute of Technology, two Micro-Electro-Mechanical System (MEMS) accelerometers are used to measure the roll angle of high-speed missile. This system puts the MEMS sensor inside the high-rotation missile [14,15]. Firstly, the system measurement equations and statistical model equations are established. Then, the improved adaptive Unscented Kalman Filter (UKF) nonlinear filtering algorithm is used to improve the accuracy of the calculation. Compared with other methods, this method emphasizes the study of the algorithm more [16].

The above methods provide many means for realizing the measurement of the roll angle of high-rotation missiles, but these methods have inevitably used large-range sensors. In the case of the existing sensor processing technology, there is a problem that the wide range sensor is not possible to meet the low-cost and high-precision requirements at the same time. To solve these problems, it is not sufficient to improve the accuracy of the solution by simply improving the algorithm. Therefore, a new flight attitude measurement system, SSINS, was used to solve these problems. Moreover, in SSINS, the key part to the range selection of the sensor that measures the roll angle is the SSP, and the rationality of its design is directly related to the accuracy of the navigation solution, because the SSP can equivalently reduce the range of the required sensor [17,18]. This structure is mounted inside the missile and used to mount MEMS inertial sensors. In contrast to SINS, the SSINS Inertial Measurement Unit (IMU) for signal acquisition is mounted on a SSP, and the SSP is not rigidly attached to the missile, but is connected to the missile via bearings, which provides a stable low-dynamic environment for the IMU and eliminates the interference of the high-speed rotation on the device’s accuracy. Therefore, the gyros with small range can be used to measure the missile’s attitude information in a relatively stable environment. In reference [17], Zhang et al. presented a measurement method for the realization of high-rotation missiles, which is a new compensation method that is proposed to remove or reduce sensor errors, so as to make it possible to maintain high precision autonomous navigation performance by MIMU when there is no external aiding. Among them, the motor is used to implement the rotation modulation method. Due to the fact that the servo motor cannot withstand a large impact, it is easily damaged or even destroyed under high overload conditions. Considering the above situation, this system may has poor stability in achieving attitude measurement in high overload environments.

In reference [18], in our lab, Duan et al. obtained the Semi-strap-down Stabilized Platform (SSP), using gravity to control the roll angular rate of the SSP. This paper analyzed and validated the dynamic model of the SSP on the ground, and proved the effectiveness of the model in the ground-based experiment. However, he did not analyze the impact of the missile’s lift force on the SSP. During the flight of a missile, due to the change of lift force, the SSP has three different states: Overweight, weightless, and normal state. The three different states have a non-negligible impact on the stability of the SSP. Therefore, in order to achieve accurate measurement of missile navigation parameters, it is necessary to consider the indirect effects of lift force on the SSP.

In the past, in order to improve the stability of the SSP, our laboratory applied many methods to reduce the friction torque of bearing. For example, in reference [19], the SSP optimization design with a dual bearing nested structure was proposed in our lab. 

Above all, in order to better replace wide-range sensors with small-range sensors, we have conducted further research. This paper describes the design and implementation of the new SSP based on the Missile-borne Semi-Strap-down Inertial Navigation System in the high-speed rotation missile-borne environment using three steps, which are described as follows. In the first step, according to the dynamic model of the stabilized platform in the ground-based experiment, the lift force of a missile is calculated when flying at 2° angle of attack, and the dynamic model of the SSP is given in the high-speed rotation missile-borne environment. The second step combines the characteristics of the space reserved inside the missile and gives the optimal design of the SSP. The last step achieves the best quality of the SSP, which can minimize the roll angular rate and roll angle of the SSP by controlling the quality of the platform. Using the Runge-Kutta method and mass gradient control method to calculate and plot the time-varying curve of the roll angle and angular rate of the SSP under different conditions, the best quality of the platform is obtained. In order to verify the above conclusions, we installed the prototype of the SSP on the flight simulation turntable and did an experiment, and the experiment results were in accordance with the theoretical results.

The remainder of this paper is organized as follows. Section 2 illustrates the working principle of the Semi-strap-down Inertial Navigation System (SSINS) and the relationship between the quality selection method of the SSP and the range of the Inertial Measurement Unit. The stability principle of the SSP is introduced and the dynamic model of the SSP in the high-speed rotation missile-borne environment is obtained. Then the SSP optimal quality selection method is proposed in Section 3. Section 4 is the implementation of the SSP, as well as test verification. The conclusion is given in Section 5.

## 2. Semi-Strap-down Inertial Navigation System 

### 2.1. The Composition and Working Principle of the SSINS

In the launch stage of the missile, assuming that the attitude of the missile is measured using the Strap-down Inertial Navigation System (SINS), the rolling axial angular rate of the SSINS will increase rapidly in a short period of time, for example, a certain missile will reach 30 r/s (10,800°/s). According to the different orthogonality of the sensor’s sensitive axis in the Micro Inertial Measurement Unit (MIMU), the angular rate’s components that the gyroscope is sensitive to will also vary in the missile’s pitch and yaw directions. In order to be able to measure the axial rotation speed of the missile, the SINS is equipped with a large-scale MEMS gyroscope, that is, a sensor with a range greater than 30 r/s. However, in the conditions of current technological level, wide-range MEMS sensors have low precision, so a large measurement error is generated in measuring the axial rotational speed of the missile. Then, we use the data with measurement error to solve the attitude of the missile, and the navigation solution error will inevitably be larger. Therefore, the accuracy of the SSINS’s angular rate measurement being too low is the most critical reason for limiting the positioning accuracy of the missile. 

**Figure 1:**
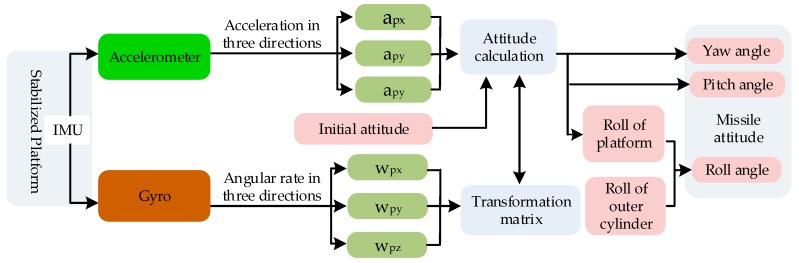
Block diagram of the overall scheme of the SMNS.

In order to solve the problem that the wide-range sensor cannot meet the requirements of high-precision navigation, the SSINS is innovatively designed based on the SINS. Figure 1 depicts the overall scheme of the Semi-strap-down Inertial Navigation System (SSINS), which is used to measure the missile navigation parameters. It can be seen from the Figure 1 that the MIMU is integrated inside the SSP. The SSINS is combined with a certain algorithm. For the convenience of description, the coordinate systems are defined as follows: The navigation coordinate system (N-frame) is chosen as the local geographical coordinate frame; the body coordinate system (B-frame) is the missile coordinate system. As the SSP equipped with an IMU is swinging in the SSINS, a new frame in which the inertial readings are collected is introduced. The new coordinate system can be referred as SSP frame (P-frame), since the IMU and the SSP are fixedly connected, so its axis is aligned with the sensitive axis of inertial sensors. Generally speaking, the navigation solution of SSINS is similar to that of conventional INS. Nevertheless, the inertial sensor outputs are collected from P-frame in SSINS. The solution algorithm is designed based on the relationship between IMU output information and missile motion parameters. As shown in Figure 1, this system can realize the measurement of most of navigation parameters through the data measured by the IMU in the SSP.

**Figure 2:**
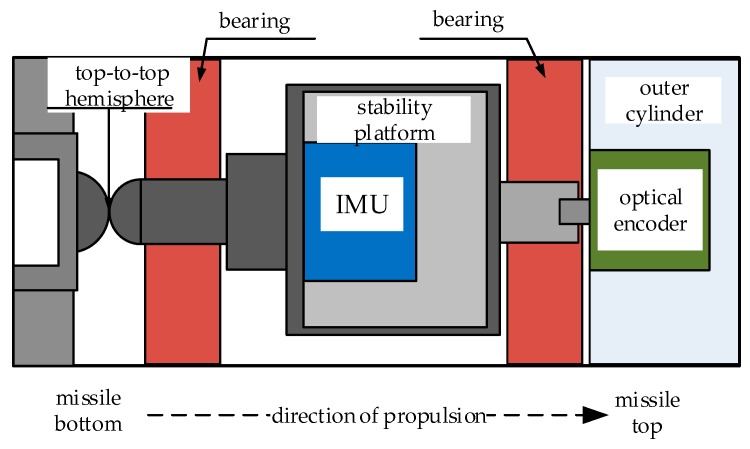
Arrangement of high precision Semi-Strapdown Inertial Navigation System (SSINS).

The specific arrangement of the SSINS is shown in Figure 2. The SSINS is mainly composed of two parts. The stabilized platform for carrying the sensor is the inner cylinder, and the rest is called the outer cylinder. An IMU is installed in the inner cylinder, and mainly contains three MEMS gyroscopes and three MEMS accelerometers. The outer cylinder shell of the SSINS is fixedly connected with the missile, and the outer cylinder is equipped with an optical-electricity encoder and a top-to-top hemisphere structure. 

In the SSINS, the photoelectric encoder is used to measure the relative rotation angle of the outer cylinder and the inner cylinder. The photoelectric encoder we used is an incremental photoelectric encoder with a resolution of 1024 P/R. The photoelectric encoder shell is fixed to the SSINS’s outer cylinder by screws, and the front part of the rotating shaft of the photoelectric encoder is semi-cylindrical, so that the photoelectric encoder’s rotating shaft can be fixedly connected with the inner cylinder, that is, both ends of the photoelectric encoder are respectively fixed to the outer cylinder and the inner cylinder. When the outer cylinder rotates synchronously with the missile, the photoelectric encoder body then rotates along with the outer cylinder, while the photoelectric encoder‘s shaft rotates following the inner cylinder, so that the relative rotation angles of the inner cylinder and the outer cylinder can be measured. After that, the relative rotation angle measured by the photoelectric encoder and the rolling attitude angle measured by gyro are summed to complete the measurement of the parameters of the missile’s rolling axis.

The SSINS has implemented overload protection in many aspects, such as top-to-top hemisphere structure. The top-to-top hemisphere structure is an axial anti overload device, the main function of which is to prevent the bearings from being damaged when the missile is subjected to overload during launch. The entire flight process of the missile can be roughly divided into three stages, namely the interior ballistic launch stage, the engine propulsion stage, and the inertial free flight stage. In order to ensure the effective work of the SSP, the top-to-top hemisphere structure is designed for the interior ballistic launch phase and the engine propulsion phase, because the overload during the above two launch phase is too large. The contact of the upper and lower parts of the top-to-top hemisphere effectively protects the bearing and ensures that the SSP can work normally. 

When the SSP is under the action of the bearing friction torque and the equivalent gravity torque, it does not completely follow the high speed rotation of the missile, and at this time the SSP is in a slightly rotated state relative to the ground. As can be seen from the above, the SSP plays a key role in the selection of the IMU range. Generally, the swing amplitude of the SSP is not particularly large, therefore a gyroscope with a range of about 200°/s can satisfy the measurement requirements in the direction of the roll axis of the SSP. In the direction of the pitch axis and the yaw axis of the missile, the gyroscope’s range only needs about 75°/s to meet the actual measurement requirements. Compared with the range of the gyroscope (its range is 10,800°/s) in the direction of the rolling axis of the SINS, the SSINS only needs to be 1/54 of this, which shows that the existence of the SSP effectively reduces the range of sensors and improves the navigation accuracy.

In conclusion, the missile is in a high-rotation state throughout the flight stage. In order to reduce the sensor range requirement in the high-speed rotation missile environment, a SSP that can isolate the high-rotational motion of the missile is designed, and the navigation solution accuracy is improved by reducing the requirement of sensor range. Nevertheless, the IMU is fixedly connected to the SSP, and the selection of the sensor’s range in the IMU is a key factor affecting the navigation accuracy of the SSINS. 

### 2.2. The Relative Position of IMU and the Choice of IMU Range. 

Micro Inertial Measurement Unit (MIMU) is the main sensor part of the SSINS. The composition of MIMU used in this system is shown as Figure 1: The yellow square represents MEMS gyro and the blue square represents MEMS accelerometer. MIMU is consisted of three mutually orthogonal MEMS accelerometers and three mutually orthogonal MEMS gyroscopes. A gyroscope and an accelerometer are mounted on one axis, but their directions are opposite. IMU is fixed within the SSP′s shell, and is used to measure the real-time angular velocity of the SSP. IMU is an information-sensitive module of inertial measurement system. The key idea of design is to ensure the sensitive axis is fit with three-dimensional orthogonal installation as much as possible. Of course, its structure should be as sturdy and compact as possible. Here, the coordinate system O_b_-R_b_Y_b_P_b_ is fixedly connected to the missile coordinate system, and the coordinate system O_p_-R_p_Y_p_P_p_ is fixedly connected to the IMU coordinate system. Lastly, R represents the roll axis, Y represents the yaw axis, and P represents the pitch axis.

**Figure 3:**
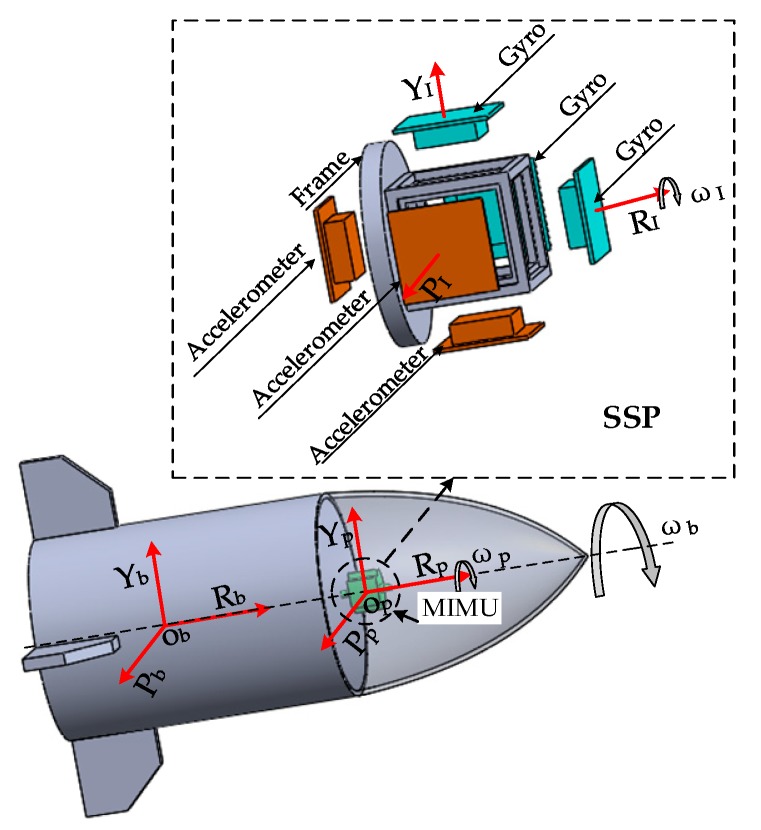
The schematic diagram of the relative position of Micro Inertial Measurement Unit (MIMU).

The schematic diagram of the relative position of MIMU is shown in Figure 3. Since the MIMU and the SSP are fixed together, the movement state of the MIMU and the SSP is the same during the flight of the missile. According to the installation method of the SSP in the missile, the gyro in the IMU can directly measure the attitude of the pitch and yaw of the missile, while the gyro of the roll axis measures the microrotation roll attitude angle of the SSP relative to the ground. Then, the microrotation roll attitude angle is summed with the relative rotation angle measured by the photoelectric encoder to obtain the roll attitude angle of the missile. The three sensitive axes of the IMU can be considered to be perpendicular to each other by calibration. The angular rates of the three sensitive axes of the IMU are quite different. For example, the roll angular rate of a certain type of missile is about 54 times the angular rate of the pitch and yaw directions. However, the installation error cannot be completely eliminated by calibration. Due to the fact that the roll angular rate is too large, a big angular velocity component is generated in the pitch and yaw axes, thereby affecting the accuracy of the missile’s navigation solution. It can be seen that reducing rotation has important significance for not only for the roll axis, but also the pitch and yaw axes.

The concept of “rotary axial isolation and radial strapdown” is realized by the SSP. In the past SSP design process, we can only select the parameters of internal sensors by experience. Moreover, there is no introduction to the selection method for the quality of the SSP in the existing materials. How to quantitatively reduce the range of MEMS inertial sensors carried in the SSP is a key issue to be solved in this article. 

In order to avoid the over-range phenomenon, the selected sensor range is often larger than the actual SSP maximum angular rate. Table 1 shows the parameters of the IMU before the SSP is optimized.

The design method and quality choice of the SSP are not only related to reducing the effect of rotation, but also related to the range of sensors we choose. The unreasonable design of the SSP not only reduces the rotational speed of the missile incompletely, but also leads to a decline in navigation accuracy by relying solely on experience to select the range. Therefore, there is an urgent need for a theoretical guide to the design method and quality choice of the SSP, to help us analyze the effect of SSP parameter selection on the isolation rotation effect and how to optimize for sensor range selection.

This section introduces a novel SSINS that uses MEMS inertial sensors to realize the navigation and positioning function of high-rotation missiles, and describes in detail how MEMS sensors and photoelectric encoders jointly measure the parameters of the missile. Since the design of the SSP determines the sensor range selection, it is necessary to start from the principle of the SSP, so that mounted the MEMS inertial sensor is better applied.

## 3. Design Principle and Optimal Quality Selection Method of SSP

### 3.1. The Introduction of SSP

#### 3.1.1. The Principle Behind the SSP

The high-speed rotation missile is a conventional missile which relies on its own rotating motion to eliminate machining errors and achieve flight stability [20], and there is a high-speed rotational motion around its own roll axis. In order to complete the high-precision navigation and positioning requirements of the missile, the accurate measurement of the roll angle cannot be completed by relying on the large-range sensor, under the condition that the existing sensor fabrication process is unchanged [21,22]. 

The SSP is a mechanical structure using for mounting sensors and creating a good working environment for MEMS inertial sensors. Its main role is to isolate the high-speed rotational motion of the missile, through the SSP to reduce the range requirements of MEMS inertial sensors, then the wide-range sensor is replaced by a small-range sensor, so it can achieve high-precision navigation and positioning. The direction of the roll axis of the SSP is connected with the missile through bearings, so the bearing friction torque and the gravity torque are the key factors of the SSP. The equivalent gravity model of the SSP is shown in Figure 3.

**Figure 4:**
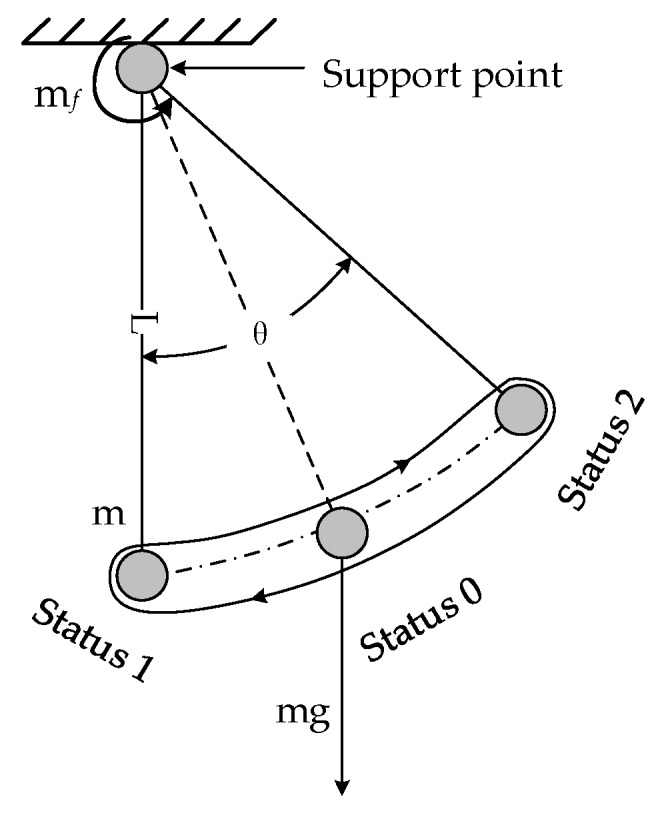
Dynamics diagram of the Semi-strap-down Stabilized Platform (SSP).

Figure 4 describes the compound pendulum motion of the SSP around its own roll axis in the case of ground tests [23,24], and it is also equivalent to the compound pendulum motion of the platform’s center of gravity around the supporting axis point in the cross-section of the platform. $m_{f}$ indicates the bearing friction torque that acts on the supporting axis point, θ represents the angle between the position of the mass point and the vertical axis, which changes with time, and L denotes an arm of force whose size is equal to the distance from the mass point to the support point. State 0 represents the equilibrium position between the bearing frictional force and the equivalent gravity moment, at this point the angular rate reaches a maximum. State 1 and State 2 indicate that the roll angular rate is zero and at this point the amplitude of the swing is greatest. The center of gravity of the stabilized platform is a pendulum movement around state 0. When the bearing friction torque $m_{f}$ is a constant value, G·L is larger, sinθ is smaller, and the lower the torque balance position, the smaller the swing amplitude of the SSP [25].

Vibrations will occur when the missile flies in the air [26,27]. In order to make the SSP undisturbed, it is necessary to increase the stability of the SSP by lowering the equivalent gravity center position of the SSP.

#### 3.1.2. Composition and Shape Design of SSP

Figure 4 shows the composition of the newly designed SSP, where part 1 represents the bearing. The front cover, the anti-overload rear cover, the upper rectangular cover, the lower arch cover, and the counterweight are represented by parts 2.1 to 2.5. Part 2.1 is fixed with the relative rotation angle measurement section, it plays a role in supporting the platform. Part 2.2 acts as an overload protection device. Parts 2.5 and 2.4 are fixed together inside, playing the role of lowering the center of gravity. The main part of the SSP is composed of parts 2.1–2.5.

**Figure 5:**
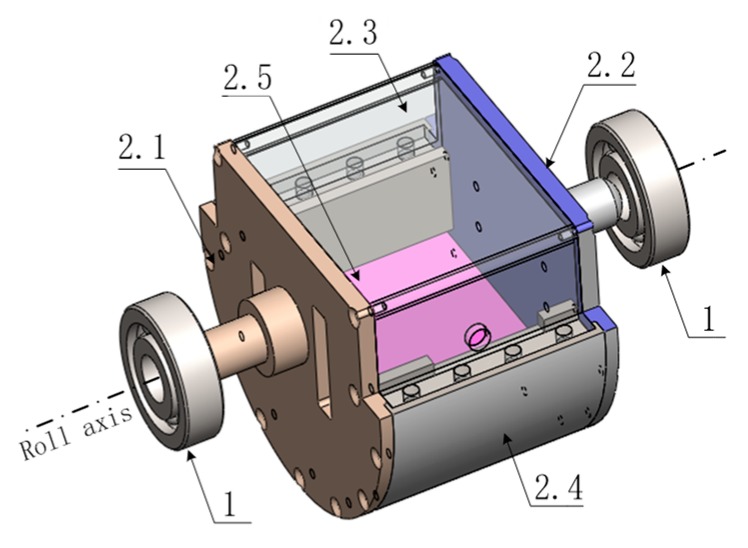
Composition diagram of the newly designed SSP.

As shown in Figure 5, the convex part of the front cover (2.1) and the back cover (2.2) are connected with the bearing inner ring though an interference fit. The bearing outer ring is fixed with the missile. When the missile rotates at a high speed, the bearing outer ring rotating with the missile at the same speed. Under the drive of the bearing friction torque, the rotation of the inner ring of the bearing causes the SSP to oscillate. At this time, the gravity torque will prevent the SSP from swinging. In order to make the SSP less prone to swinging, we need to reduce the center of gravity of the SSP. According to the spatial characteristics of the cylinder space reserved in the missile, the novel shape structure shown in Figure 5 is designed.

In order to make maximum use of the space inside the missile and reduce the center of gravity of the SSP, the novel shape of the SSP is designed as shown in Figure 5. The measuring device is light in weight and can be neglected, so the position of the center of gravity of the SSP is determined by the parts of the shell composed of parts 2.1 to 2.4, and the counterweight indicated by part 2.5. The position of the center of gravity of the SSP′ shell can be derived from the following equation:

(1) $$\frac{\iint y\cdot \rho_{i}ds}{\iint \rho_{i}ds}$$

where $\rho_{i}$ and y denote the density of different materials and the axis of symmetry of the SSP. The SSP shell part consists of two materials. The material of the upper rectangular cover 2.3 is duralumin, and the other parts are alloy steel. The material of the upper rectangular cover 2.3 is duralumin, its main function is to reduce the quality of the upper part of the SSP. The remaining part of the shell is made of alloy steel, whose role is to increase the quality of the bottom of the SSP, and it can also resist the overload, so it can protect the IMU inside the SSP. Because the materials of the shell are different, the center of gravity is calculated according to the two different parts. The center of gravity of the above two parts is deduced by Equation (1), and the center of gravity of the shell can be obtained according to the two points of gravity center position equation. The equation for the two-point center of gravity is as follows:

(2) $$\left\{ \begin{array}{l} w=\frac{C_{c}\cdot x_{1}+C_{n}\cdot x_{2}}{C_{c}+C_{n}}\\ t=\frac{C_{c}\cdot y_{1}+C_{n}\cdot y_{2}}{C_{c}+C_{n}} \end{array}\right.$$

where $C_{c}$ denotes the center of gravity of upper rectangular cover and $C_{n}$ describes the center of gravity of the three parts 2.1, 2.2, and 2.4; $C_{c}\left(x_{1},y_{1}\right)$ and $C_{n}\left(x_{2},y_{2}\right)$ represent the above two parts of the center of gravity, M(w,t) represents the equivalent center of gravity position of the shell part. The relevant data involved in the calculation are shown in Table 2.

The position of the center of gravity of the shell is around (0, 10 mm). In order to increase the force arm of the SSP′s gravitational restoring moment by reducing the position of the center of gravity of the SSP, a counterweight with a large density is placed at the bottom of the SSP near part 2.4. As the quality of the counterweight increases, the parameters such as the quality of the SSP, the arm of force, the moment of inertia, and the frictional torque of the bearing will change. Therefore, we can change the parameters of the SSP by controlling the quality of the counterweight. 

### 3.2. Dynamic Model of the SSP in Missile-borne Environment

#### 3.2.1. Force Acting on the Missile

**Figure 6:**
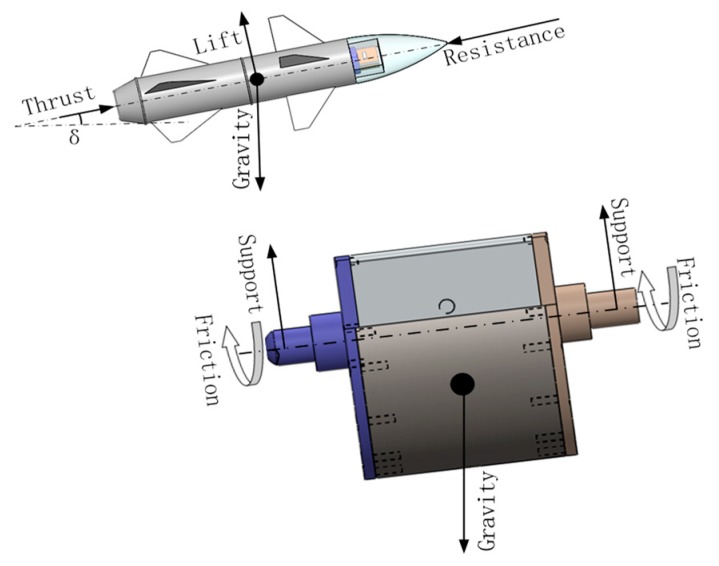
Diagram of main forces acting on missile and the SSP.

During the missile’s flight in the air, it is subject to forward thrust from the direction of the missile’s rolling axis, resistance in the opposite direction of the missile’s velocity, lift perpendicular to the missile’s roll axis, and vertical downward gravity. If the missile’s aerodynamic configuration, speed, and angle of attack are determined, the lift of the missile is certain [28]. The upper left corner of Figure 6 is the force diagram of the missile in the missile-borne environment, and the lower right corner is the force diagram of the SSP under the same conditions.

In Figure 6, it can be seen that the forces acting on the SSP are mainly friction force of bearing, gravity, and supporting force, when the high-rotation missile’s flight remains in a stable state. In the vertical direction, there is acceleration of the missile, since the SSP is a part of the missile, it has the same state of motion as the missile, so they have the same acceleration in the vertical direction. According to the different acceleration of the missile in the vertical direction, the SSP is in an overweight state, a weightless state, or a normal state. When the missile has an upward acceleration during the flight, in the direction of the missile’s vertical rolling axis, the lift force of the missile is greater than gravity, and the SSP is in an overweight state. At this moment, the effect of gravity and acceleration is consistent to prevent the SSP from swinging and the stability effect is enhanced. When the missile has downward acceleration during the flight, in the direction of the missile’s vertical rolling axis, the lift of the missile is less than gravity. At this time, the SSP is in a weightless state, the stability of the platform is weakened at this moment. When the missile’s acceleration in the vertical direction during the flight is zero, that is, the same as the ground test, only gravity acts as a stabilizing effect on the platform [29].

In summary, according to the different lift force of the missile, the stability of the SSP is also different. The SSP′s dynamic model previously proposed in our lab is a special case where the acceleration of the missile is zero in the vertical direction. It does not fully consider the impact of changes in lift force on the stability of the SSP in the actual missile-borne environment. This article innovatively analyzes the characteristics of a certain missile’s lift for the first time, and fully considers the influence of the change of the missile’s vertical direction lift on the SSP, and establishes a dynamic model of the SSP under the actual missile-borne environment for the first time.

#### 3.2.2. Lift Calculation of a Certain Missile

During the missile’s flight, due to the relative movement between the missile and the air, the missile is subjected to forces from the air [30]. The lift force acting on the missile can be calculated by Equation (3):

(3) $$F_{l}=\frac{1}{2}\rho \cdot S\cdot C_{y}\cdot V^{2}$$

where $\rho =1.293\;kg/m^{3}$ denotes air density, S denotes the maximum cross-sectional area of the missile, V denotes the speed of the missile relative to the air, and $C_{y}$ denotes the lift force coefficient combined with the characteristics of missile structure, which can be calculated by Equation (4):

(4) Cy=Cncosδ−CAsinδ={2δ−0.0063δ[1−(DbD)2]+4Cxt(lcd+ltd)sin2δπ}−{Cx0+1.51+lnd[2δ−0.0063δ(DbD)2]}sinδ

where $C_{n}$ describes the normal-force coefficient of the missile, $C_{A}$ describes the missile’s axial force coefficient, δ describes the attack angle of the missile (in radians), D indicates the bottom diameter of the missile,$D_{b}$ denotes the diameter of the missile, $l_{c}$ describes the length of the cylinder, $l_{t}$ indicates the tail length of the missile, d describes the maximum diameter of the missile, and $l_{n}$ describes the length of the missile head. When the flow around the projectile is a laminar flow boundary layer, $C_{xt}=1.2$. When the flow around the projectile is a turbulent flow boundary layer, $C_{xt}=0.35$. $C_{x0}$ represents zero drag coefficient and can be solved by Equation (5):

(5) $$\begin{array}{ll} C_{x0} & =C_{xf}+C_{xb}+C_{xw}\\ & =C_{xf}+1.14\frac{d_{b}^{4}}{ld^{3}}\left(\frac{2}{Ma}-\frac{d_{b}^{2}}{l_{d}}\right)+\left(0.0016+\frac{0.002}{Ma^{2}}\right)\left(\psi_{0}^{1.7}+a_{k}^{1.7}\sqrt{1-\frac{d_{b}^{2}}{d}}\right) \end{array}$$

The missile will be affected by different types of air resistance depending on the speed of flight. These air resistances are frictional resistance, eddying resistance, and shock wave drag [31]. $C_{xf}$ represents the frictional resistance coefficient of the missile. $C_{xb}$ represents the eddying resistance coefficient of the missile. $C_{xw}$ represents shock wave drag coefficient of the missile. Equation (5) is further described as the following according to the flight characteristics of a missile.

(6) $$\begin{array}{ll} C_{x0} & =C_{xf}+C_{xb}+C_{xw}\\ & =C_{xf}+1.14\frac{d_{b}^{4}}{ld^{3}}\left(\frac{2}{Ma}-\frac{d_{b}^{2}}{l_{d}}\right)+\left(0.0016+\frac{0.002}{Ma^{2}}\right)\left(\psi_{0}^{1.7}+a_{k}^{1.7}\sqrt{1-\frac{d_{b}^{2}}{d}}\right)\\ & =\frac{0.072\mu^{0.2}}{{\left(\rho vl\right)}^{0.2}}\frac{S_{s}}{S}\frac{1.2}{\sqrt{1+0.2{\left(Ma\right)}^{2}}}+1.14\frac{d_{b}^{4}}{ld^{3}}\left(\frac{2}{Ma}-\frac{d_{b}^{2}}{l_{d}}\right)+\left(0.0016+\frac{0.002}{Ma^{2}}\right)\left(\psi_{0}^{1.7}+a_{k}^{1.7}\sqrt{1-\frac{d_{b}^{2}}{d}}\right) \end{array}$$

In Equation (6), $d_{b}$ stands for the bottom diameter of the missile; l is the maximum length of the missile; Ma is Mach number; $\psi_{0}$ is cone half-angle of the missile (in radians); $\alpha_{k}$ is the cone angle of the missile tail (in radians); and μ is viscosity coefficient of air and its value is $1.8\times 10^{-5}$ pa/s. When Equations (4)–(6) are substituted into Equation (3), we can get a lift force when the angle of attack is δ. The parameters used to calculate the lift force can be seen in Table 3.

**Figure 7:**
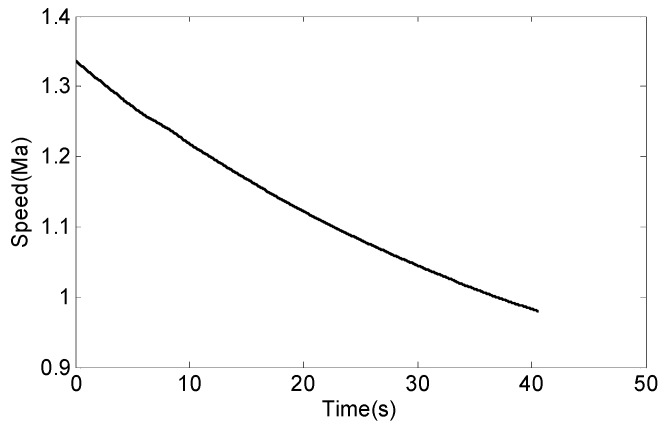
The speed of the missile.

Assume that the attack angle of the missile is constant and the value is 2°. The missile’s flying speed is shown in Figure 7. We can get the trend of missile lift force with time.

We can determine from Figure 7 that the lift of the missile is between 150 N and 300 N.

#### 3.2.3. Establishment of Dynamic Model in Missile-Borne Environment

In the missile-borne environment, the lift force and the component of gravity act together in the direction of the roll axis perpendicular to the missile, and the resultant force provides the acceleration for the missile in this direction [32]. The SSP and the missile have the same motion state in the pitch and yaw directions, because the SSP is fixedly connected to the missile in the above directions. In other words, the SSP has the same acceleration as the missile in the direction of the roll axis that is perpendicular to the missile. The resultant forces of gravity components and supporting force of the SSP provides the acceleration for the SSP in the direction of the roll axis that is perpendicular to the missile; the supporting force of the SSP is provided directly by the bearing. The direction of the supporting force acting on the SSP and the lift force acting on the missile are the same, and there is a relationship between the magnitude of the two forces. The specific relationship can be obtained by the following two equations:

(7) $$\left\{ \begin{array}{l} m_{m}g\cdot \cos\delta -F_{l}=m_{m}a\cdot \cos\delta \\ m_{p}g\cdot \cos\delta -F_{s}=m_{p}a\cdot \cos\delta \end{array}\right.$$

In Equation (7), $m_{p}$ represents the quality of the SSP; δ represents the angle of attack; $F_{s}$ represents the supporting force of the SSP; a represents the acceleration acting in the direction of the roll axis that is perpendicular to the missile; $m_{m}$ represents the quality of the missile; and $F_{l}$ represents the lift force of the missile. The relationship between the supporting force of the SSP and the lift force of the missile can be described as Equation (8):

(8) $$F_{s}=\frac{m_{p}}{m_{m}}\cdot F_{l}$$

From Equation (8), it can be seen that when the lift force and quality of the missile are determined, the supporting force of the SSP is proportional to the quality of the SSP.

The effect of the component of gravity which is perpendicular to the roll axis of the SSP can be divided into two parts. The part of gravity that interacts with the support force of the SSP plays a role in increasing the stability of the SSP, so the value of this part of gravity of SSP is equal to the value of the support force. The effect of the one part of gravity and $F_{s}$ constitutes an interaction force, since they are equal and opposite. Another part of gravity provides acceleration which is perpendicular to the roll axis of the SSP. According to Ref. 5, the dynamic model of the SSP in the ground condition is shown as Equation (9):

(9) $$M_{f}+L\cdot F_{s}\cdot \sin\theta \left(t\right)\cdot \cos\delta =J_{p}\overset{\cdot \cdot }{\theta }\left(t\right)$$

In Equation (9), $M_{f}$ represents friction torque of bearing; $J_{p}$ represents a moment of inertia of SSP; and θ represents the angle between the arm of gravity of the SSP and the vertical direction. According to Equations (8) and (9), the dynamic model of the SSP in the missile-borne environment can be obtained.

(10) $$M_{f}+L\cdot \frac{m_{p}}{m_{m}}\cdot F_{l}\cdot \sin\theta (t)\cdot \cos\delta =J_{p}\overset{\cdot \cdot }{\theta }$$

When the angle of attack δ of the missile is constant, then cosδ is a constant value. It can be seen from Equation (10) that in order to minimize the swing angular rate of the SSP, this can be achieved by setting the ratio of $M_{f}$ to $L\cdot \frac{m_{p}}{m_{m}}\cdot F_{l}\cdot \sin\theta (t)$ as one. Under the premise that the swing angle of the SSP is the smallest, in order to minimize the swing amplitude of the SSP the weight of the $L\cdot \frac{m_{p}}{m_{m}}\cdot F_{l}$ part must be increased. In summary, in order to minimize the swing angular rate and swing angle of the SSP, we can find the $m_{p}$ which minimizes the $\frac{M_{f}}{L\cdot F_{s}}$ value by controlling the $m_{p}$ value by combining Equations (9) and (10). In addition, we can see that the angular acceleration of the rolling axis is independent of the change of the g value during the flight of the missile. Therefore, by controlling the quality of each part of the SSP, the range of the MEMS sensor for measuring the angular rate of rolling is reduced.

This section takes a certain type of missile as an example to obtain the equation for describing the change of the SSP′s roll angular rate in the missile-borne environment, and clarifies the variation law of the roll angular rate of SSP; we can, through the study of the law of roll angular rate, optimize the selection of the range of MEMS sensors installed in the SSP.

### 3.3. The Method of Selecting the Best Quality of the SSP

#### 3.3.1. Design Objectives of the SSP

The SSP based on the missile-borne Semi-Strap-down Inertial Navigation System is a mechanical structure that is installed in a high-speed rotation missile, and its main function is to mount the MEMS sensor and create a good working environment for the MEMS inertial sensor, so the stability of the SSP will be directly reflected in the MEMS inertial sensor range requirements [33]. The better the stability of the SSP, the lower the range requirement of the sensor and the higher the measurement accuracy of the navigation parameters. In other words, the ultimate aim of the SSP design is to reduce the roll angular rate and improve the accuracy of navigation positioning. 

#### 3.3.2. The Relationship between Bearing Friction Torque and Supporting Force on the SSP

With the increase in the SSP′s quality and the bearing pressure increase, it leads to an increase in bearing friction torque. The relationship between the bearing pressure and the friction torque of the bearing can be calculated by the bearing friction torque equation [34]. Equation (11) is the Svenska Kullager-Fabriken (SKF) bearing friction torque equation:

(11) $$M_{f}=G_{rr}{\left(v\cdot n\right)}^{0.6}+G_{sl}\cdot \mu_{sl}+M_{seal}+M_{drag}$$

Bearings were strictly selected, as the Semi-strap-down Inertial Navigation System (SINS) uses an open bearing. Therefore, there is no bearing friction torque $M_{seal}$ caused by the sealing element. The bearing frictional torque $M_{drag}$ caused by oil drag loss, eddy-current, and splashing accounts is only a small fraction of the total, so in this article, we do not calculate it. The expressions of rolling friction torque coefficient $G_{rr}$ and sliding friction torque coefficient $G_{sl}$ are related to bearing type and axial load. Their expressions are definite when the bearing type and axial load are determined. In this paper, we take deep groove ball bearing 6200 as an example to study the best quality of the SSP. Bearing friction torque is only related to axial pressure and radial pressure when bearing type is determined. When the axial pressure of the bearing is zero, the rolling friction torque coefficient and sliding friction torque coefficient can be expressed by the following equation:

(12) $$\left\{ \begin{array}{l} G_{rr}=R_{1}\cdot d_{m}{}^{1.96}\cdot F_{r}{}^{0.54}\\ G_{sl}=S_{1}\cdot d_{m}{}^{-0.26}\cdot F_{r}{}^{\frac{5}{3}} \end{array}\right.$$

In Equation (12), $R_{1}$ and $S_{1}$ are bearing-related geometric constants which can be obtained by referring to the bearing catalogue. $d_{m}$ represents the average diameter of the bearing. $F_{r}$ represents the radial pressure of bearing. Because two bearings are installed on the SSP, the radial pressure of each bearing is 1/2 of the SSP gravity. Then Equation (12) can be further described as:

(13) $$M_{f}=0.5^{0.54}R_{1}\cdot d_{m}{}^{1.96}\cdot {\left(m_{p}g-m_{p}a\right)}^{0.54}{\left(\nu \cdot n\right)}^{0.6}+0.5^{\frac{5}{3}}\cdot S_{1}\cdot d_{m}\cdot {\left(m_{p}g-m_{p}a\right)}^{\frac{5}{3}}\cdot \upsilon_{sl}$$

In Equation (13), (a) describes the acceleration of the missile in the vertical direction. When the radial pressure $F_{a}$ of the bearing is greater than zero, the rolling friction torque coefficient and sliding friction torque coefficient of the bearing are as follows:

(14) $$\left\{ \begin{array}{l} G_{rr}=R_{1}\cdot d_{m}{}^{1.96}{\left(F_{r}+\frac{R_{2}}{\sin\alpha_{F}}\cdot F_{a}\right)}^{0.54}\\ G_{sl}=S_{1}\cdot d_{m}{}^{-0.145}\cdot {\left(F_{r}{}^{5}+\frac{S_{2}\cdot d_{m}{}^{1.5}}{\sin\alpha_{F}}\cdot F_{a}{}^{4}\right)}^{\frac{1}{3}}\\ \alpha_{F}=24.6\cdot {\left(F_{a}/C_{0}\right)}^{0.24} \end{array}\right.$$

In Equation (14), $C_{0}$ indicates a bearing basic steady load. When $F_{a}$ is not zero, the relationship between the friction torque of the bearing and the quality of the stabilized platform can be determined by Equation (7), as shown in Equation (15):

(15) $$\begin{array}{ll} M_{f} & =R_{1}\cdot d_{m}{}^{1.96}\cdot {\left[0.5\frac{m_{p}}{m_{m}}\cdot F_{l}\cdot \cos\delta +\frac{R_{2}}{\sin\alpha_{F}}\cdot 0.5\cdot \frac{m_{p}}{m_{m}}\cdot F_{l}\cdot \sin\delta \right]}^{0.54}\cdot {\left(\nu \cdot n\right)}^{0.6}+\\ & S_{1}\cdot d_{m}{}^{-0.145}{\left[0.5^{5}\cdot {\left(\frac{m_{p}}{m_{m}}\cdot F_{l}\right)}^{5}\cdot \cos^{5}\delta +\frac{S_{2}d_{m}{}^{1.5}}{\sin\alpha_{F}}\cdot 0.5^{4}{\left(\frac{m_{p}}{m_{m}}\cdot F_{l}\right)}^{4}\cdot \sin^{4}\delta \right]}^{\frac{1}{3}}\cdot 0.05 \end{array}$$

In Equation (15), δ represents the angle between the roll axis of the missile and the horizontal line. $R_{2}$ and $S_{2}$ are bearing-related coefficients which can be obtained by referring to the bearing catalogue. ν is the coefficient related to the bearing type. n is the relative angular rate of the inner and outer rings. The angular rate of the inner ring is small and can be negligible. In the following calculations, n can be approximately equal to the roll angular rate of the missile.

#### 3.3.3. The Choice of the Best Quality Interval for the SSP

**Figure 8:**
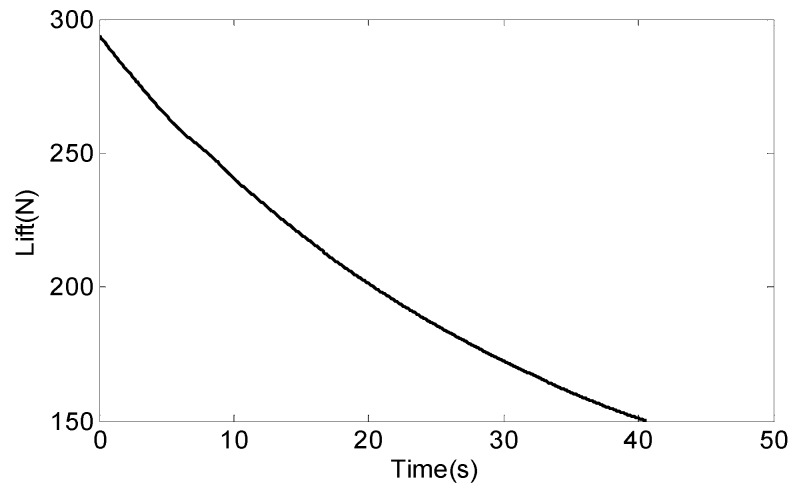
The lift force missile.

In order to increase the stability of the SSP and reduce the angular rate of the SSP in the direction of the roll axis, we have to study the variation of the value of $M_{f}/\left(L\cdot F_{s}\right)$ according to Equation (9). We find the $M_{f}/\left(L\cdot F_{s}\right)$ value that makes the SSP angular rate minimal by controlling the quality of the SSP. Form the above analysis, it is known that the supporting force and effective gravity of the SSP are interacting forces. The change rule between the value of $M_{f}/\left(L\cdot F_{s}\right)$ and the quality of the SSP can be obtained through simulation, then the optimal quality of the SSP can be found when the ratio is the minimum. Since the axial pressure being zero belongs to a special case, this situation is not considered in the actual analysis. Therefore, in actual analysis, the relationship between the bearing friction torque and the quality of the SSP is expressed by Equation (14). The relevant data is substituted into Equation (14) to obtain the change rule of the ratio of bearing friction torque to the quality of the SSP with $m_{p}$ when the attack angle of the missile is δ. Since a missile flies at an attack angle of 2°, the missile lift force curve of Figure 8 is combined, and we take the lift force of 100 N, 150 N, 200 N, and 250 N, respectively. 

The above parameters are brought into Equation (15), then the simplified Equation (16) is obtained when the attack angle is 2°.

(16) $$\begin{array}{l} \frac{M_{f}}{L\cdot F_{s}}=\frac{0.0105}{F_{s}}{\left[0.0454F_{l}\cdot m_{p}+0.2777\cdot m_{p}\cdot \frac{F_{s}}{\sin(0.1919\cdot m_{p}{}^{0.24})}\right]}^{0.54}\\ +\frac{1.046\cdot 10^{-4}}{L\cdot F_{s}}\cdot {\left[1.9345\cdot 10^{-7}{\left(m_{p}\cdot F_{l}\right)}^{5}+2.0674\cdot 10^{-8}\frac{{\left(m_{p}\cdot F_{l}\right)}^{4}}{\sin(0.1919\cdot m_{p}{}^{0.24})}\right]}^{\frac{1}{3}} \end{array}$$

**Figure 9:**
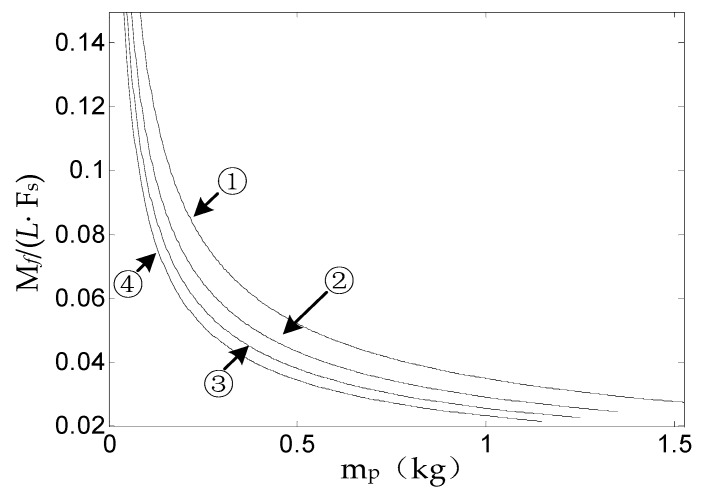
Changing curve of the ratio of $M_{f}/(L\cdot F_{s})$ following $m_{p}$.

Figure 9 describes, when the axial load of the bearing is not zero, the variation between the ratio of the bearing friction torque to the support force of the SSP and the quality of the SSP. The curves labeled with the numbers ①, ②, ③, and ④ indicate the case where the lift is 100 N, 150 N, 200 N, or 250 N, respectively.

As can be seen from Figure 8, the ratio of $M_{f}/(L\cdot F_{s})$ is inversely proportional to the quality of the SSP. In order to minimize the angular rate of the SSP, we find the quality of the SSP when the ratio of $M_{f}/(L\cdot F_{s})$ is minimized. According to Figure 8, the best quality of the SSP is greater than 1 kg.

### 3.4. Optimization Design of the SSP

Assume that the missile is subject to a lift of 200 N, we change the quality of the counterweight to observe the trend of the roll angle and the maximum angular rate of the SSP with the quality. When the lift force of the missile is 200 N, the support force and bearing friction torque of the SSP can be obtained by solving Equations (8) and (14), respectively. The SSP′s support $F_{s}$ and bearing friction torque $M_{f}$ are brought into Equation (9), so that Equation (9) is transformed into a second-order differential equation about α(t). The required parameters can be found in Table 4 and Table 5.

The data in Table 5 are the simulation data obtained from the SolidWorks. $m_{pz}$ represents the quality of counterweight. Bearing friction torque can be calculated according to Equation (15). Bring the relevant data in Table 5 into Equation (8), and divide $J_{p}$ to the left of the equation. The equation is solved by the Runge-Kutta method and the curve is drawn. Equation (17) can be obtained, after the first row of data in Table 4 is brought into Equation (9).

(17) $$y_{2}=83.28-250.523\cdot \sin(y_{1})$$

In Equation (17), $y_{2}$ is the SSP roll angular rate, and $y_{2}$ is the roll angle of the SSP. Equation (17) is solved by using the Runge-Kutta method, and the curve of the angular rate and roll angle of the SSP is drawn. In this paper, Equation (17) is solved and the diagrammatic drawing is created when the quality of the SSP is 1029.97 g, 1492.67 g, 1681.18 g, and 1875.09 g respectively. 

**Figure 10:**
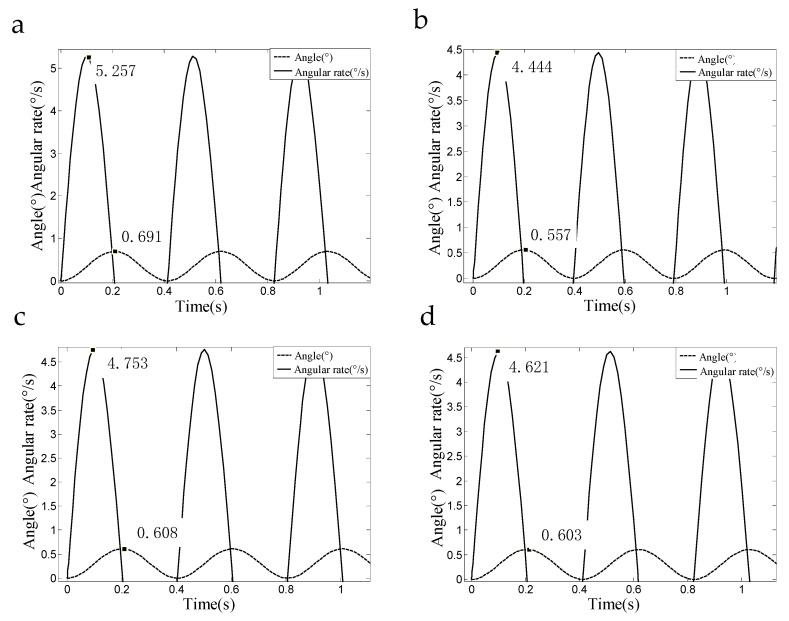
Curves of the roll angular rate and the roll angle of the SSP change with the quality: (**a**) A curve of roll angular rate and roll angle vary with time when the quality of the SSP is 1029.97 g; (**b**) A curve of roll angular rate and roll angle vary with time when the quality of the SSP is 1492.67 g; (**c**) A curve of roll angular rate and roll angle vary with time when the quality of the SSP is1681.18 g; (**d**) A curve of roll angular rate and roll angle vary with time when the quality of the SSP is1875.09 g.

Table 6 shows the variation of the maximum roll angular rate and the maximum roll angle of the SSP when the quality of the SSP changes. As can be seen from Figure 10 and Table 6, with increasing quality of the SSP, the angular rate and the roll angle of the SSP exhibit the same trend. It can be seen from Table 6 that when the quality of the SSP is about 1492 g, the roll angular rate is the smallest, and the stability of the SSP is best at this time. That is to say, the range of the inertial MEMS sensor mounted on the SSP is greatly reduced, and the navigation accuracy of the SSINS is improved.

## 4. Test Verification

**Figure 11:**
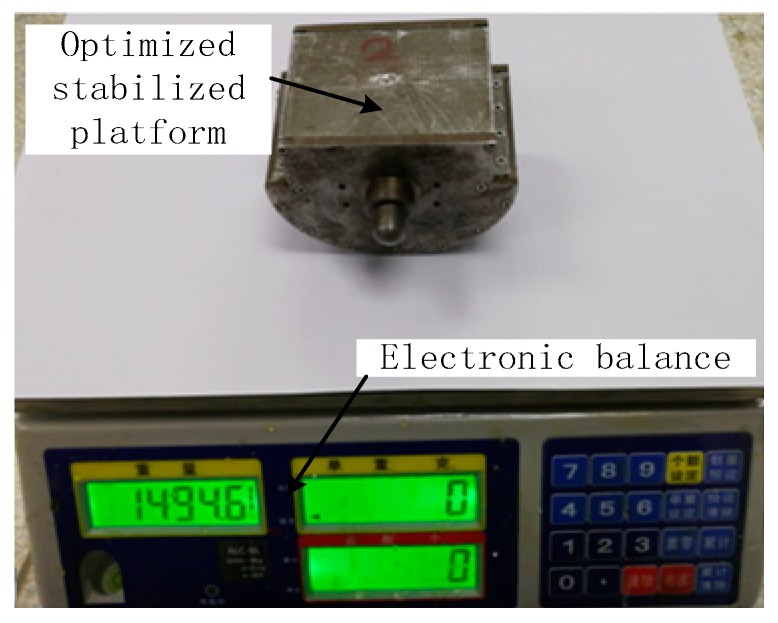
Quality of the SSP after optimization.

**Figure 12:**
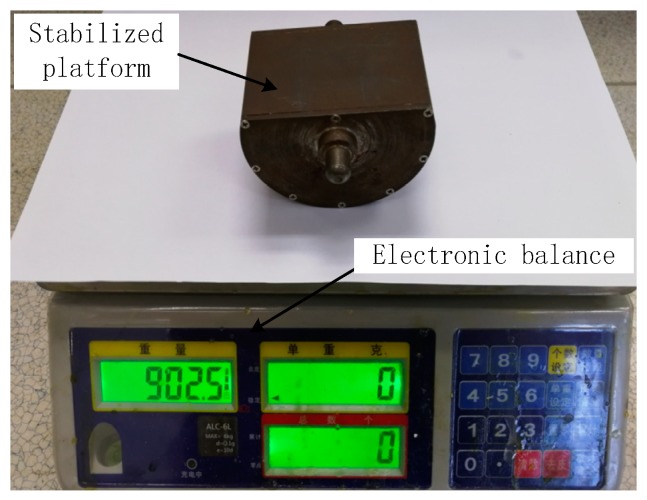
Quality of the SSP before optimization.

According to the above analysis, the quality of each part of the SSP was strictly controlled, and the quality of the final integrated SSP is 1494.6 g. The quality of the optimized SSP and the unoptimized SSP is shown in Figure 11 and Figure 12, respectively. It is obvious that the quality of optimized SSP is larger than the unoptimized SSP.

A number of shooting tests were conducted by using the unoptimized system and the optimized system. Test results showed that the measurement precision of the optimized system is higher than the unoptimized system. Due to the variability of the shooting test conditions, the superiority of the optimized system cannot be fully explained. According to the above, when the attack angle of the missile is 2°, the lift force of the missile is greater than gravity. In this condition, the stability of the SSP is better than that of the ground test. Therefore, the ground test can be used to verify the stability of the new SSP. In order to verify the isolation effect of the SSP to the high-speed rotational motion of the missile and the correctness of the theoretical analysis, a SSINS with SSP was used to perform the experiments by using a high-precision tri-axial flight simulator. The tri-axial flight simulator has three rotational frames, namely, outer frame, middle frame, and inner frame. Table 7 and Table 8 summarize the technical parameters of tri-axial flight simulator and the characteristics of the IMU in the optimized SSINS, respectively.

The comparison of the parameters of the sensors in the two tables in Table 1 and Table 8 shows that the gyro on the roll axis is reduced by ± 150°/s through the optimized design range of the SSP, and the performance parameters of the sensor are also improved. Due to the decrease in the roll angular rate of the SSP, the centrifugal force of the *X* and *Y* axes is reduced, so the *X*, *Y* axis acceleration measurement range is changed from 2.5 g to 0.85 g. It can be seen from the above that the small range sensor’s error is smaller than the larger one, so the use of a small range sensor effectively helps to improve the navigation accuracy.

In order to verify the effect of the SSINS before and after optimization, the comparison experiment is performed under the same experimental conditions. First, the isolation rotation comparison experiment is performed to compare the tri-axial angular rate of the SSINS before and after the optimization. Then, the comparison experiment of the roll angle calculation accuracy is carried out. The roll attitude angle measured before and after the optimization was compared with the feedback value of the flight simulation turntable roll angle, and the two difference values were compared.

The experiment conditions are set as shown in Table 9. In the above two experiments, the SSINS’s pitch and yaw angle provided by high-precision tri-axial flight simulator are same. 

**Figure 13:**
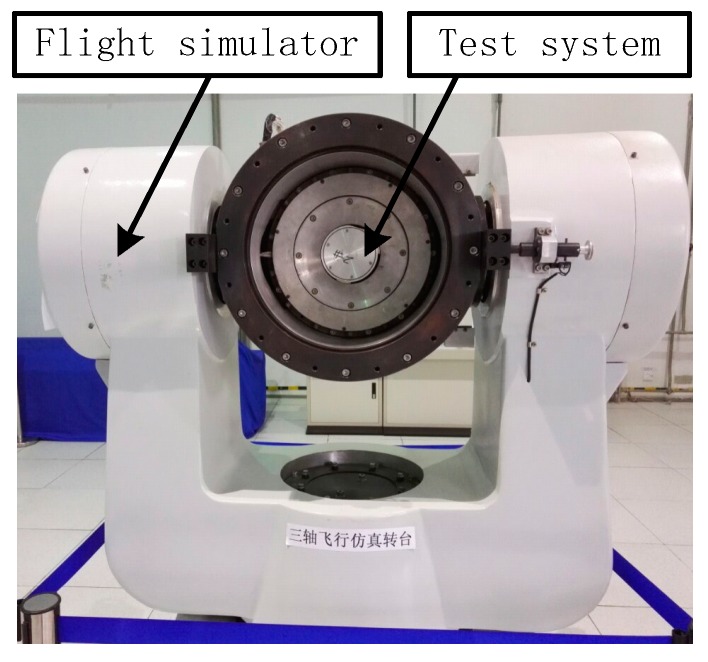
Flight simulator test.

**Figure 14:**
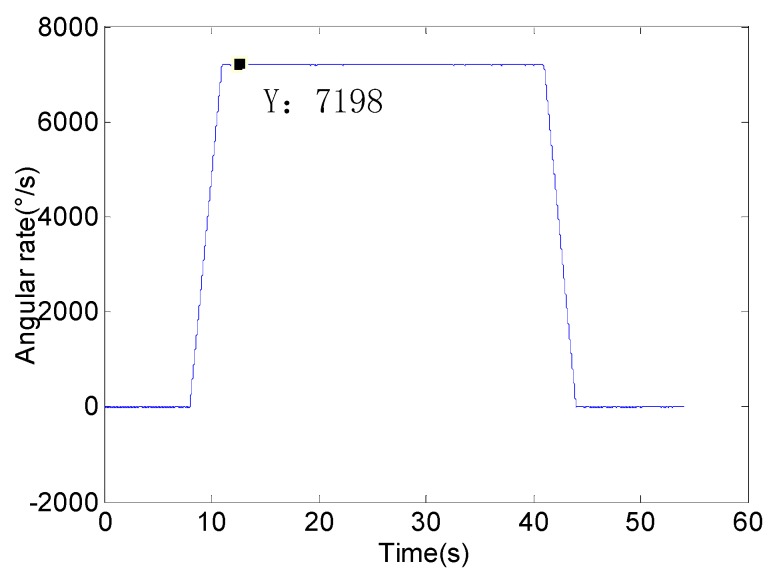
Feedback angular rate of flight simulation turntable.

The unoptimized system and the optimized system were installed on the flight simulator and the experiments were carried out. The scene of the flight simulation experiment is shown as Figure 13. The experiment conditions are set as Table 9. We set the rotational speed of the flight simulator is 20 r/s and the pitch angle is 2°. The feedback angular rate of flight simulation turntable is shown in Figure 14. The system experiment data is solved by the same procedure and SSP attitude information can be obtained. 

**Figure 15:**
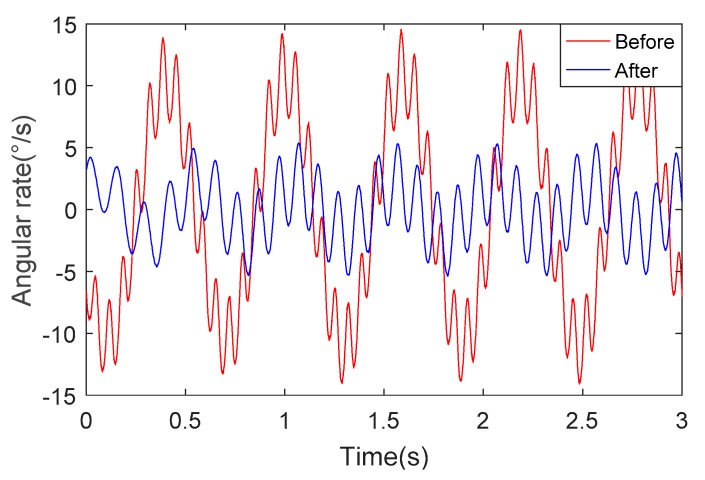
Angular rate in the direction of the roll axis.

Figure 15 depicts the change of roll angular rate for the unoptimized and optimized systems under the same experimental conditions. We can determine from Figure 15 that the roll angular rate of the SSP after optimization is about 1/3 of the angular rate of the SSP before optimization. This shows that the SSP after optimization has better stability and ability to isolate the high-rotational motion of the missile body.

**Figure 16:**
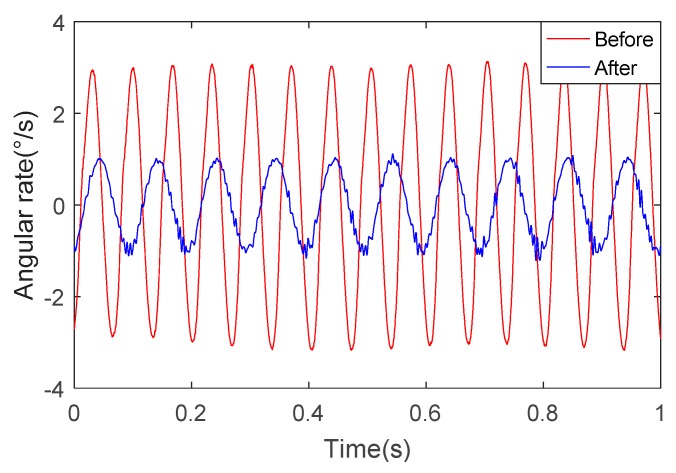
The angular rate of the pitch axis.

**Figure 17:**
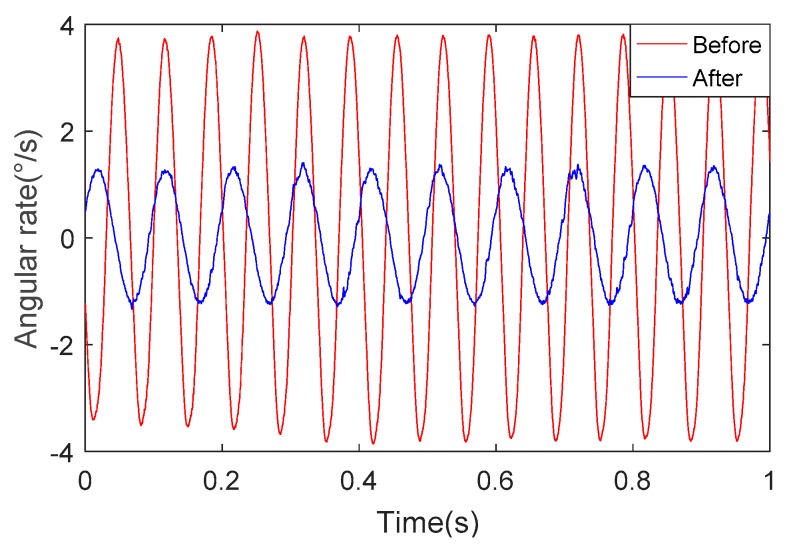
The angular rate of the yaw axis.

We can see from Figure 16 and Figure 17 that the angular rate of the SSP after optimization is about 1/3 of the angular rate of the SSP before optimization. This once again proves the superiority of the optimized SSP.

**Figure 18:**
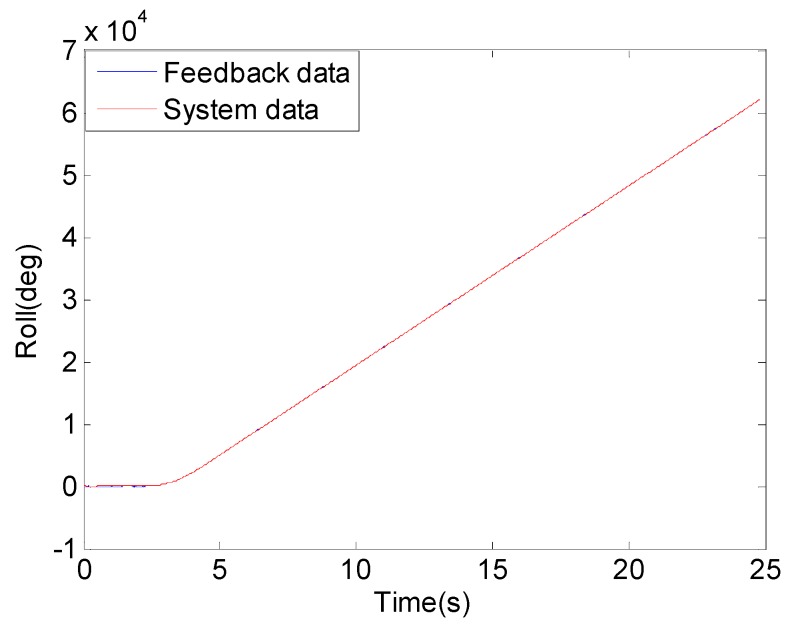
Roll attitude.

**Figure 19:**
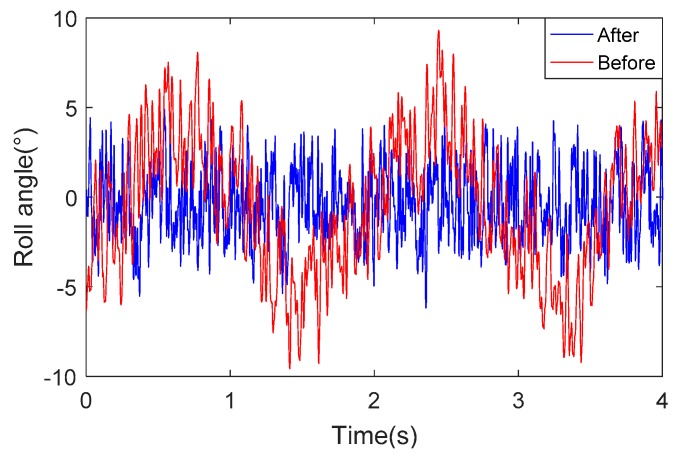
Rolling attitude angle difference.

Figure 18 describes the change in roll angle over time, the blue curve represents the feedback value of roll angle of flight simulation turntable and red curve represents the measured roll angle of the optimized system. We can see from the Figure 18 that the change trend of the two curves is the same. Figure 19 shows the difference between the roll angle measured by the system and the roll angle of the flight simulator. The red curve represents the difference between the roll angle measured by the system before optimization and the feedback value of the roll angle of the flight simulator. The blue curve represents the difference between the roll angle measured by the system after optimization and the feedback value of the roll angle of the flight simulator. After comparison, it was found that since the SMINS used a small range sensor after optimization, the error of the solution was reduced to 60% of the system that used a wide range sensor. Through the flight simulation turntable experiment, the practicability and effectiveness of the proposed method in actual application was illustrated. 

## 5. Conclusions

The Semi-Strapdown Inertial Navigation System (SSINS) is a new type of system that can measure the navigation parameters of high-speed rotational missiles. In this paper, taking a typical missile type as an example, the lift force curve of this missile was calculated for the first time. The author innovatively proposes a dynamic model of the SSP in the missile-borne environment, which has greater practical application value. In addition, firstly, based on the new shape of the SSP, we controlled the quality of the SSP, then looked for the relationship between the quality of the SSP and the force affecting the roll angular rate from the principles of the SSP. Finally, when the angle of attack of the missile is constant, the SSP′s quality is obtained by the Runge-Kutta method when the swing angle and the swing angle rate are minimized. The choice of the best quality method proposed for the first time is based on the mechanism of the SSP, which enables the MEMS inertial sensor to measure the attitude of the missile in a relatively stable environment with high precision. The experiment results show that the measured roll angle error is reduced to 60% of the unoptimized measurement. It provides a new method for attitude and position measurement of high-rotation and high-overload missiles. Furthermore, there is no systematic theoretical study on the design principle and quality selection method of the SSP based on the SSINS. The first proposed quality selection method provides theoretical guidance for the selection of the range of MEMS inertial sensors in the SSP. 

## Figures and Tables

**Table 1:** Parameters of the Inertial Measurement Unit (IMU) before the Semi-strap-down Stabilized Platform (SSP) is optimized.

Characteristics		Range	Bias	Random Walk
	Gyroscope (*X* axis)	±200°/s	12°/h	0.2${}^{\circ}/\sqrt{Hz}$
	Gyroscopes (*Y*, *Z* axis)	±75°/s	12°/h	0.2${}^{\circ}/\sqrt{Hz}$
	Accelerometer (*X* axis)	±10 g	0.75 mg	150 ug/$\sqrt{Hz}$
	Accelerometer (*Y*, *Z* axis)	±2.5 g	0.75 mg	150 ug/$\sqrt{Hz}$

**Table 2:** Parameters related to stable platform.

Parameters’ Name	Value
Internal diameter R_1_ (mm)	36
External diameter R_2_ (mm)	40
Thickness H (mm)	4
Length L (mm)	64
Width K (mm)	23.5
The density of steel $\rho_{1}$ (g/mm^3^)	7.85 × 10^−3^
The density of lead $\rho_{2}$ (g/mm^3^)	11.34 × 10^−3^

**Table 3:** Parameters of the missile.

Parameters	Value
l	0.690 m
$l_{c}$	0.258 m
$l_{n}$	0.325 m
$l_{t}$	0.095 m
$S_{s}$	0.0658 m^2^
d	0.125 m
$d_{b}$	0.080 m
$\psi_{0}$	0.1889 rad
$\alpha_{k}$	0.5236 rad

**Table 4:** Parameters of bearing.

Parameters’ Name	Value
R_1_	3.9 × 10^−7^
R_2_	1.7
S_1_	3.23 × 10^−3^
S_2_	36.5
$d_{m}$ (mm)	20
$m_{m}$ (kg)	22
$\theta_{1}$ (°)	2
ν (mm/s)	68
N (r/min)	20

**Table 5:** Simulation Parameters.

$m_{p}$ (g)	$m_{pz}$ (g)	L (mm)	J (kg∙mm^2^)	$F_{z}$	$M_{f}$ (N·mm)
1029.97	518	19.16	715645.36	9.363	0.0596
1195.17	684	19.33	796875.50	10.865	0.0653
1311.25	800	19.17	858230.70	11.921	0.0662
1370.82	859	19.1	889924.76	12.462	0.0712
1431.32	920	18.97	923317.63	13.012	0.0732
1492.67	981	18.69	957770.86	13.570	0.0753
1554.81	1043	18.55	993279.38	14.135	0.0774
1617.67	1106	18.32	1029732.48	14.706	0.0795
1681.18	1170	18.01	1067066.03	15.283	0.0813
1745.3	1234	17.65	1105219.02	15.866	0.0816
1809.95	1298	17.41	1144145.55	16.454	0.0837
1875.09	1363	17.18	1183755.35	17.046	0.0858

**Table 6:** The maximum angular rate and the maximum angle of the SSP with different quality.

$m_{p}$ (g)	The Amplitude of the Angular Rate (°/s)	The Angle of Magnitude (°)
1029.97	5.277425125	0.6913055894
1195.17	5.401864992	0.6841432462
1311.25	4.742293892	0.5968631809
1492.67	4.444337347	0.55772058380
1617.67	4.789094705	0.6084000808
1681.18	4.752837345	0.6082058380
1745.30	4.464310826	0.5752954076
1875.09	4.621437347	0.6033058380

**Table 7:** Technical parameters of tri-axial flight simulator.

Position Accuracy (°)	Rotation Rate Accuracy (°/s)	Rotation Rate (°/s)		
		Inner Frame	Middle Frame	Outer Frame
0.001	0.001	0.001–12,000	0.001–400	0.001–400

**Table 8:** Characteristics of IMU in the optimized Semi-Strapdown Inertial Navigation System (SSINS).

Characteristics	Range	Bias	Random Walk
Gyroscope (*X* axis)	±50°/s	0.12°/h	0.017${}^{\circ}/\sqrt{Hz}$
Gyroscopes (*Y*, *Z* axis)	±25°/s	0.1°/h	0.015${}^{\circ}/\sqrt{Hz}$
Accelerometer (*X* axis)	±10 g	0.75 mg	150 ug/$\sqrt{Hz}$
Accelerometer (*Y*, *Z* axis)	±0.85 g	0.75 mg	50 ug/$\sqrt{Hz}$

**Table 9:** Setting of the experiment conditions.

	Pitch	Yaw	Roll	Rotating Mechanism
Experiment 1	+2 deg	0 deg	20 r/s	Before
Experiment 2	+2 deg	0 deg	20 r/s	After
